# Phasor‐Based Spatio‐Spectral Segmentation for Hyperspectral Fluorescence Microscopy

**DOI:** 10.1002/jbio.70351

**Published:** 2026-09-03

**Authors:** Maciej Barna, Grazyna Palczewska, Grzegorz Soboń, Krzysztof Palczewski, Jakub Bogusławski

**Affiliations:** ^1^ Laser & Fiber Electronics Group, Faculty of Electronics, Photonics and Microsystems Wrocław University of Science and Technology Wrocław Poland; ^2^ Department of Ophthalmology and Visual Sciences, Gavin Herbert Eye Institute—Brunson Center for Translational Vision Research University of California Irvine Irvine California USA; ^3^ Department of Physiology & Biophysics University of California Irvine California USA; ^4^ Department of Chemistry University of California Irvine California USA; ^5^ Department of Molecular Biology and Biochemistry University of California Irvine California USA

**Keywords:** clustering, multiphoton microscopy, ophthalmology, phasor, phasor analysis, unmixing

## Abstract

Fluorescence microscopy is essential for visualizing cellular and tissue structures. When combined with a hyperspectral modality, it enables the acquisition of emission spectra that provide insight into the composition of fluorophores. These spectra can be used for fluorophore‐based image segmentation; however, accurate spectral separation becomes challenging when fluorophore emission spectra overlap and mix within individual pixels. We present a spatially‐corrected density peak clustering (SC‐DPC) method that combines phasor transformation with histogram‐based density‐peak detection and additional spatial correction to achieve accurate and fast clustering. SC‐DPC effectively handles large datasets and clusters that are not separable linearly in phasor space while maintaining high clustering accuracy and computational efficiency. Results with synthetic data and experimental multiphoton microscopic data from the mouse retina demonstrate that SC‐DPC provides a robust, high‐performance approach for hyperspectral fluorescence data segmentation.

## Graphical abstract

Spatially‐corrected density peak clustering (SC‐DPC) enables high‐accuracy segmentation of hyperspectral fluorescence microscopy data in the presence of significant spectral and spatial overlap between fluorophores. Validated on retinal imaging, SC‐DPC effectively separates overlapping fluorophore spectra in photon‐starved conditions, offering superior robustness and speed compared to traditional algorithms like k‐means and GMM.

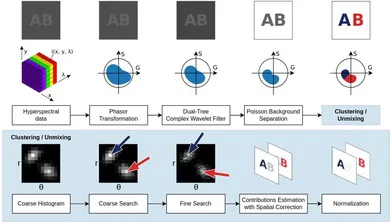

## 1 Introduction

Fluorescence microscopy is a widely used imaging technique in biomedical research, including ophthalmology [1], dermatology [2], neuroscience [3], and plant research [4]. It has demonstrated significant utility in applications such as imaging cancerous tissues and differentiating them from healthy tissue [5]. Several advanced techniques can further enhance fluorescence microscopy, such as multiphoton excitation, which enables deeper penetration with reduced risk of sample damage, enabling in vivo imaging and the observation of dynamic processes in living organisms [6, 7].

The spectral characteristics of fluorescence emission can be exploited to analyze the biochemical composition and functional state of biological samples. Despite many advances, conventional fluorescence microscopy is typically limited to a small number of spectral channels, restricting its ability to resolve complex biochemical environments. Particularly, in autofluorescence imaging, multiple endogenous fluorophores often contribute simultaneously, leading to significant spectral overlap. Hyperspectral fluorescence microscopy overcomes this limitation by acquiring a full emission spectrum at each pixel and, when combined with multiphoton excitation, it is well suited for in vivo tissue imaging [8]. However, the presence of multiple fluorophores within single pixels often leads to highly mixed spectra, making accurate separation of fluorophores challenging.

To address this problem, several algorithms, such as k‐means, non‐negative matrix factorization (NMF), and others, have been used for spectral‐based data segmentation. While these approaches are mature and effective in fields like satellite imaging and remote sensing, they face significant limitations when applied to photon‐limited fluorescence microscopy [9, 10, 11]. In biomedical fluorescence imaging, and particularly in autofluorescence, the primary challenge is often a photon‐starved signal with a strong noise presence induced by scattering in the sample, fluctuations of excitation‐light intensity, and other factors. Because spectral unmixing is often a non‐convex and ill‐posed problem, many approaches rely on iterative optimization [12]. In these low‐photon regimes, such optimization becomes highly sensitive to initialization and prone to converging to suboptimal local minima [13, 14]. Furthermore, the combination of a low signal‐to‐noise ratio with the spatial and spectral mixing of multiple fluorophores within single pixels requires additional constraints and severely degrades segmentation accuracy [15]. These challenges collectively have made accurate and efficient hyperspectral unmixing in biomedical applications a complex problem and have motivated the development of alternative approaches.

Phasor analysis is an approach that has gained widespread use in fluorescence imaging, including applications in live‐cell analysis, hyperspectral microscopy, and the characterization of complex biological environments; it provides an intuitive representation of fluorescence data [16, 17, 18, 19]. The widespread adoption of the phasor method is largely due to its ability to simplify the analysis of complex spectral data. In this approach, the spectrum from each pixel is transformed into a single point in a two‐dimensional phasor plot, where pixels with common spectral properties form distinct groups that relate to fluorophores present in the sample [16, 20]. Moreover, the phasor approach performs dimensionality reduction, since each spectrum is converted into just three values, namely, the *G* and *S* components that describe the position in the phasor space, and the additional integrated intensity *I*. The phasor approach has been successfully applied in various biomedical fluorescence‐imaging applications [21]. Despite its widespread adoption, relatively little research has been conducted on the automated analysis of spectral phasor data [16, 22]. Proposed solutions struggle with unmixing when a high number of fluorophores are present or when they mix within single pixels. Sometimes additional clusters need to be created to represent the composition of multiple fluorophores.

In this work, we present a novel clustering algorithm, spatially‐corrected density peak clustering (SC‐DPC), designed to segment fluorescence data containing multiple fluorophores with significant spectral overlap. Implementing corrections based on spatial relations between neighboring pixels in the integrated intensity image created by integrating photon counts from all spectral channels enables nonlinear clustering, improving its accuracy. The proposed workflow consists of three main steps: phasor transformation, two‐stage filtration, and clustering performed with the SC‐DPC algorithm, which allows faster processing and improves clustering accuracy in the presence of multiple spatially and spectrally overlapping fluorophores. This approach addresses the initialization issues (described above) by replacing the iterative optimization approach with histogram‐based analysis and additional spatial correction, while maintaining high accuracy in the presence of fluorophores with significant spectral overlap. Moreover, it scales well for larger datasets and produces easily interpretable results, simplifying further analysis. While the primary objective of SC‐DPC is robust clustering, it also enables the quantitative estimation of intensity contributions from multiple spectral components within a single pixel (unmixing). The workflow presented in this report was first evaluated on synthetic hyperspectral data and then applied to experimental data. For experimental validation, we selected a particularly challenging dataset, two‐photon–excited fluorescence imaging of the mouse retina, characterized by low photon counts per pixel, measurement noise, and broad, strongly overlapping fluorophore emission spectra [23]. SC‐DPC was compared with k‐means, which remains one of the most widely used clustering algorithms for phasor‐based fluorescence‐data analysis in biomedical applications [22, 24, 25, 26]. Furthermore, SC‐DPC was compared with the Gaussian‐mixture‐model (GMM) algorithm [27] to validate the unmixing results. To distinguish the influence of denoising from the clustering method itself, the analyses by k‐means, GMM, and SC‐DPC were applied to the same filtered data.

## 2 Spatially‐Corrected Density Peak Clustering (SC‐DPC)

### 2.1 Workflow

**FIGURE 1:**
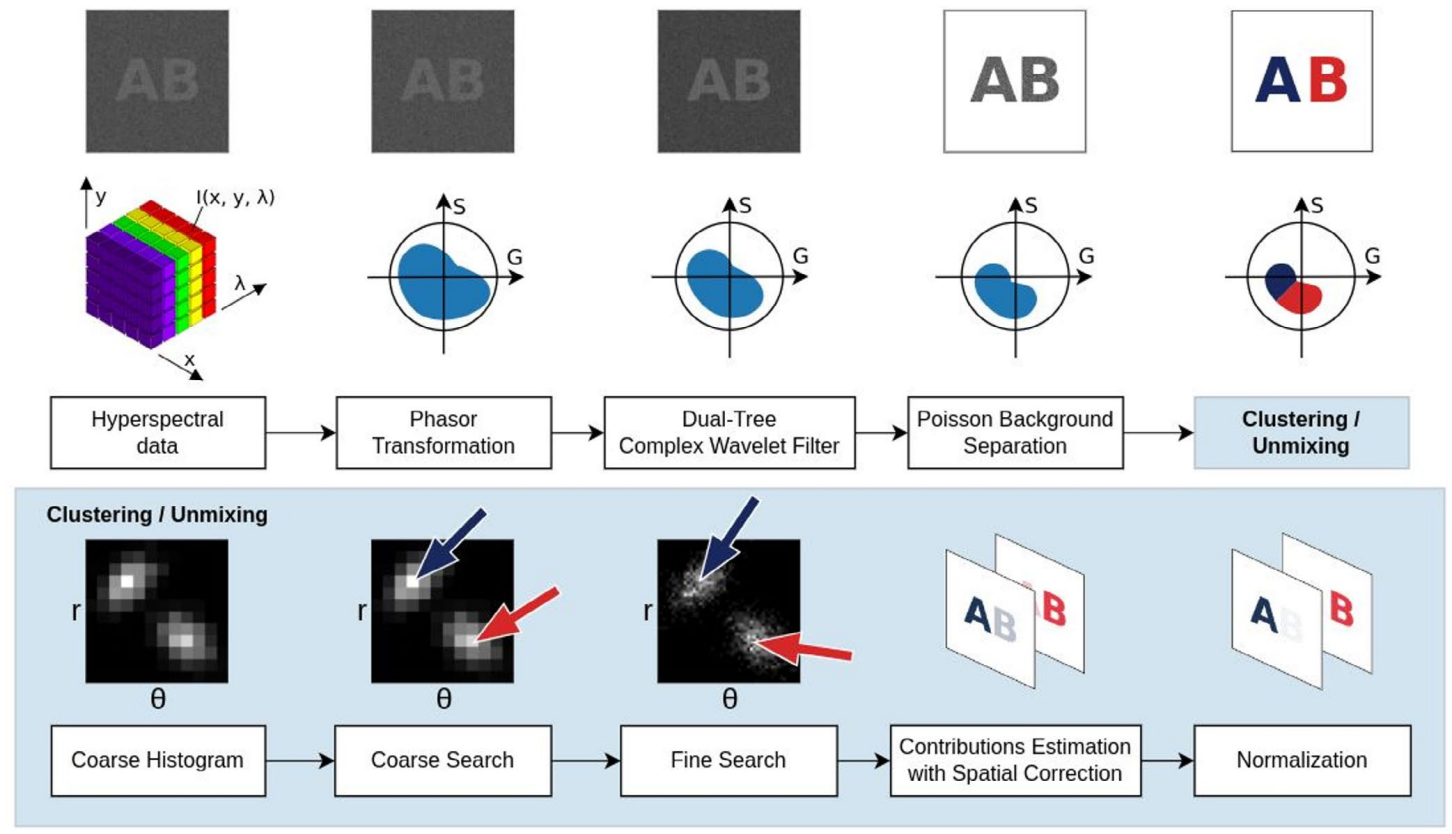
Visualization of the data‐processing pipeline. The pipeline includes hyperspectral data acquisition, phasor conversion, filtering, and clustering or unmixing stages used in this work. The first row shows changes in the intensity image containing letters A and B, transitioning from a mixed grayscale state to unmixed fluorophores (blue and red). The second row shows the corresponding processing in phasor space, where points are ultimately colored according to the dominant fluorophore (blue and red colors correspond to two distinct fluorophores). The sub‐steps of clustering/unmixing are presented in the blue frame below the main pipeline. The histograms used in this step are constructed from the polar coordinates of phasor points (*θ*, *r*).

The processing procedure for hyperspectral data analysis is conceptually presented in Figure 1. The top workflow outlines the general pipeline, starting with data acquisition or synthesis. Next, the phasor transformation converts spectra from individual pixels into 2D points on a polar plot [16, 20, 21]. Afterward, two filtering procedures are performed. First, the dual‐tree complex wavelet filter (DT‐CWF) [28] removes photon shot noise from each phasor point. Second, Poisson background separation (PBS) [29] divides all pixels into signal and background groups based on their intensity distribution. In the final step, phasor points representing pixels labeled as signal are subjected to clustering or unmixing, which is conceptually expanded in the bottom workflow of Figure 1. The first three steps (constructing a polar histogram from the phasor points, followed by a coarse search and fine search) identify *n* density peaks (cluster centers) that represent the base spectral components. Once these cluster centers are identified, initial contributions are estimated and refined using the localized spatial‐correction term. Finally, the contributions are normalized to sum to 1. The resulting values represent the continuous unmixed fractions; however, they could also be screened relative to a threshold to isolate only the dominant fluorophore per pixel and represent a discrete clustering output.

### 2.2 Phasor Transformation of Hyperspectral Data

Hyperspectral data are stored in the form of a 3D tensor, where two dimensions correspond to spatial coordinates (*XY*) and the third corresponds to wavelength (*λ*). A spectrum registered in any pixel is a vector along the *λ*‐axis, while a cross‐section at any given *λ* forms a 2D image representing the fluorescence intensity registered within a selected spectral channel. Phasor transformation uses the first sine and cosine components of a Fourier transform of spectra recorded in individual pixels [16, 20, 21]. It is given by Equations (1) and (2):

(1) $$G=\frac{\sum_{\lambda }I\left(\lambda \right)\cos\left(\frac{2\mathrm{\pi n\lambda}}{L}\right)}{\sum_{\lambda }I\left(\lambda \right)},$$

(2) $$\begin{array}{c} S=\frac{\sum_{\lambda }I\left(\lambda \right)\sin\left(\frac{2\mathrm{\pi n\lambda}}{L}\right)}{\sum_{\lambda }I\left(\lambda \right)}, \end{array}$$

where *I*(*λ*) is the intensity of the fluorescence at wavelength *λ*, *n* is the number of the harmonic (in our case always 1), and *L* is the width of the spectral band. Each spectrum in individual pixels is converted into a point at (*G*, *S*) coordinates in a 2D plane. Both coordinates take values from −1 to 1. The angular position of the point relates to the center of mass of the spectrum, and the radial position relates inversely to its width. Processing hyperspectral tensors can be computationally intensive, especially for a larger number of spectral channels. The data volume can be reduced by applying phasor transformation, which reduces volume from *X* × *Y* × *N*_*λ*_ data points, where *N*_*λ*_ describes the number of spectral channels, down to *X* × *Y* × 2, since each phasor point is represented by two coordinates, *G* and *S*.

### 2.3 Data Filtration

Data filtration was performed in two steps, first on phasor points and second on intensity data. The first step aimed to remove photon shot noise from each pixel, represented by a phasor point, using the DT‐CWF described in [28]. We used the implementation presented in [30], adjusted to our data with two levels of wavelet decomposition. The second step focused on dividing the pixels into two groups labeled as background and signal, based on the distribution of their intensities. This step assumes that noise follows a Poisson distribution, and the signal does not, so by comparing the mean of all pixel intensities with their variance, the presence of a signal can be detected. If the Poisson criterion is not satisfied, the highest‐intensity pixel is labeled as a signal and moved into the signal group. Then, the mean and the variance of the remaining set of pixels are recalculated and compared again. The process continues until the variance of the unlabeled pixels matches their mean within a margin (standard deviation (SD) multiplied by *k*, set to 1 by default); that is, the Poisson criterion is satisfied. Those pixels were labeled as background, and together with their corresponding phasor points, they were later excluded from clustering. The pixels moved into the signal group were retained and were subjected to clustering. The division process was performed using the PBS method described in [29]. This step is particularly important for photon‐starved datasets, where the mean photon count per pixel is on the order of tens of photons. Without background removal, low‐intensity noise pixels would bias the clustering outcome. While the proposed number of decomposition levels and the value of *k* parameter are robust, they can be modified to adjust the filtering strength.

### 2.4 Clustering and Unmixing

In this work, we designed a novel algorithm, SC‐DPC, for accurate phasor data segmentation without sacrificing efficiency. The objective of our algorithm is to identify cluster centers representing base fluorophores in a phasor plot, and estimate their intensity contributions in each pixel. The algorithm consists of three main stages: density peak detection in a histogram created from the phasor plot, spatially corrected estimation of intensity contributions, and final normalization.

The first stage uses a graph‐based search on the phasor density histogram to find a user‐specified number of density peaks *n*, which represent the number of fluorophores. If *n* is unknown, an iterative exploratory approach should be adopted, starting with an estimated number based on prior knowledge of the sample and preliminary inspection of the phasor‐plot shape. When working with a labeled sample, *n* should be set to the number of labels. It can be further incremented by 1 to account for a significant autofluorescence background that filtering stages did not fully suppress. By establishing parent–child relationships between neighboring histogram bins, where parent is the bin with a higher number of phasor points inside, the algorithm identifies bins without parents as local density maxima. If the initial search returns more local maxima than *n*, Gaussian smoothing is applied to reduce the surplus of spurious peaks. Any unmodeled components are merged into the *n* detected clusters without corrupting the positions of their centers. While the underlying graph‐based search is based on the algorithm described in [31], we modified it by introducing a coarse‐to‐fine search strategy to stabilize peak detection against spurious density peaks. Specifically, this approach first finds approximate peak regions using a low‐resolution histogram and then conducts a high‐resolution search within those regions to locate the cluster centers with high precision. For the coarse search, both angular and radial histogram bin counts were selected to satisfy the Nyquist‐Shannon sampling criterion for the 10‐nm spectral channel width to avoid binning artifacts. If prior knowledge is available regarding the minimum spectral separation between pure components or the width of their spectra, the bin sizes can be adjusted to smooth out peaks related to finer features. For the subsequent fine search, the bin sizes can be chosen arbitrarily to achieve desired accuracy. More details on this stage and the algorithm used are provided in the Supporting Information.

The second stage of SC‐DPC calculates the contributions of the detected peaks for every phasor point. The initial values depend on the distance between a phasor point and each peak, which are then refined using a localized spatial‐correction term based on the pixel's 3 × 3 neighbors in the integrated intensity image. Established spatially regularized unmixing approaches [12] rely on probabilistic frameworks (e.g., Markov Random Fields), and on total variation regularizers, or global‐regularization terms requiring iterative solvers. In contrast, SC‐DPC is deterministic and localized, and operates entirely on distance geometry. The regularization expression evaluates the distance of the central pixel relative to all cluster centers, alongside the distances of the immediate eight spatial neighbors to those same centers. The resulting weight acts as a localized group “vote” across the nine pixels, biasing the estimated contributions toward cluster with stronger representation among neighboring pixels. This approach stabilizes the weights of noisy pixels in uniform image regions and enforces spatial consistency in regions where clusters overlap in phasor space, without any additional knowledge about the image structure or its statistical properties. The SC‐DPC algorithm was named after this operation.

Finally, the contributions for each pixel are normalized so they sum to 1. While these fractions can be scaled according to the total intensity of each pixel to find the partial intensities expressed in photon counts, in the following sections fluorophore contributions are expressed as a percentage of each pixel's total intensity (normalized fractions multiplied by 100%).

While SC‐DPC inherently performs unmixing, its output can be simplified to identify a single “dominant” fluorophore, providing clustering of phasor points. This conversion allows for a direct comparison with traditional clustering methods like k‐means and enables the flexibility to perform either clustering or unmixing, depending on the specific needs of the analysis.

## 3 Methods

### 3.1 Synthetic Data

**FIGURE 2:**
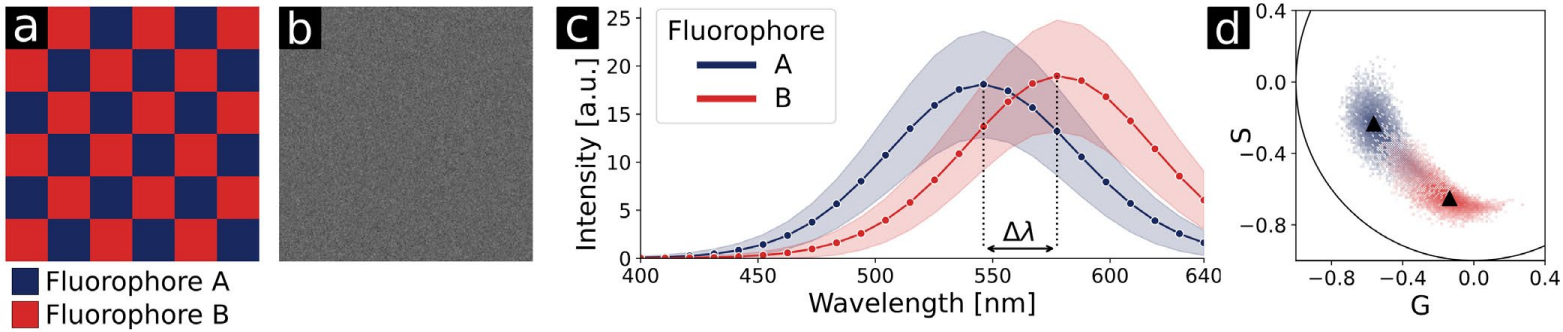
Example of a synthetic dataset. (a) Ground‐truth spatial distribution of dominant fluorophores; (b) intensity image representing an integral of photon counts from all spectral channels; (c) statistical distribution of the pure spectra generated for the two fluorophores, where solid lines denote the mean intensity, and shaded regions represent one SD (spectral separation Δ*λ* is set to 40 nm; 50% of pixels contain mixed intensity contributions derived from these base spectra); (d) phasor representation of the dataset, where each point is marked with a color corresponding to the dominant fluorophore. Black triangles indicate the cluster centers, defined as the mean positions of all points belonging to the cluster.

Synthetic data used for evaluating the SC‐DPC method were generated by first constructing an image with a chessboard pattern representing the ground‐truth spatial distribution of two fluorophores, A and B (Figure 2a). This pattern was then converted into an intensity image, representing an integral of photon counts from all spectral channels, with the mean total intensity set to 100 photon counts per pixel (Figure 2b). We note that the location of fluorophores was indistinguishable in the intensity image. Hyperspectral data were produced by assigning a Gaussian spectrum to each pixel, centered at the corresponding peak wavelength. To make the simulation more realistic, noise following a Gaussian distribution was added independently to the spectral center of mass and spectral width. This provides a controlled way to simulate variability in spectra and their mixtures. Figure 2c illustrates an example of the statistical distribution of the pure spectra generated for the two fluorophores, where solid lines denote the mean intensity, and shaded regions represent one SD. In this example dataset, the pure spectra were mixed in 50% of the pixels to simulate the mixing of fluorophores. More physically realistic noise simulation approaches, such as Monte Carlo‐based modeling of photon detection, have been presented [32] and may be used in future work. To further increase similarity with the experimental data, the generated spectra were truncated on the longer‐wavelength (red) side. Truncation occurs when the observed spectral range is not wide enough to capture the entire emission spectrum, which is often a case in two‐photon excited autofluorescence imaging and may lead to skewed results. The combination of spectral truncation and noise added to spectral parameters caused phasors to exhibit elongated tails toward longer wavelengths. The image included pixels containing spectra from individual fluorophores, along with pixels exhibiting mixed contributions from both fluorophores in random proportions. During clustering, pixels containing mixed contributions were classified according to the dominant fluorophore; for example, a pixel with 70% of its intensity from fluorophore A and 30% from fluorophore B was assigned to group A. Finally, the hyperspectral data were transformed into phasor form using the first harmonic within the 400–640 nm range. Figure 2d shows the resulting phasor plot, with each point colored according to its dominant fluorophore, and the same color‐coding is mapped back onto the original image to visualize the spatial localization of the dominant fluorophore within each pixel. It is important to indicate that the phasor points form two overlapping clouds, whose shapes depend on the characteristics of the added noise, the degree of fluorophore mixing, and the spectral truncation. To illustrate how spectral truncation affects the distribution of phasor points, we have generated an additional untruncated dataset, which is presented in Figure S1.

### 3.2 Experimental Hyperspectral Two‐Photon Excited‐Fluorescence Data

The experimental data used in this work, which were previously presented in [23], consist of an ex vivo hyperspectral two‐photon excited‐fluorescence image of RPE cells from an albino *Abca4* ^*−/−*^ *Rdh8* ^*−/−*^ mouse. The data were obtained with an instrument based on the Leica TCS SP8 Falcon equipped with a Chameleon Vision S laser, photon counting hybrid photodetectors, and a 20×, 1.0 NA, 1.95‐mm working distance objective. A mode‐locked Ti:sapphire laser (Coherent Chameleon) was used as the excitation source, tuned to a 730‐nm central wavelength. Laser light, ranging up to 5 mW, was directed into the mouse's eye throughout the imaging process. Spectral data were acquired using the integrated spectral‐detection system of the Leica microscope. In this method, the spectral components of the fluorescent light were spatially separated by a prism, then isolated by a motorized slit, and detected by a hybrid photodetector in a de‐scanned configuration. For each pixel in the image, its fluorescence‐emission spectrum was recorded and divided into 24 wavelength channels, ranging from 400 to 640 nm, with a 10‐nm bandwidth per channel. The total acquisition time for each dataset was ~31 s, with 1.3 s per single wavelength channel.

### 3.3 Animals and Study Approval

All mice were housed in the animal facility in the Laboratory Animal Resources (ULAR) Center at the University of California, Irvine, where they were maintained on a normal mouse‐chow diet under complete darkness, or in a 12‐h light (∼10 lx)/12‐h dark cyclic environment. Mice were anesthetized with an intraperitoneal injection of ketamine (20 mg/mL) and xylazine (2 mg/mL) diluted with water at a dose of 5 μL/g body weight. Before imaging, the mouse pupils were dilated with 1% tropicamide. Animal procedures were approved by the Animal Care Committees at the University of California, Irvine (UCI) and conformed to the recommendations of both the American Veterinary Medical Association Panel on Euthanasia and the Association for Research in Vision and Ophthalmology under assurance no. A‐3416‐01. Albino *Abca4* ^*−/−*^ *Rdh8* ^*−/−*^ double‐knockout mice were generated as previously described [33, 34]. The mice used in this study were 2‐ to 4‐month‐old.

### 3.4 K‐Means

The k‐means clustering algorithm [35] was used in this work as a reference to evaluate the clustering performance of the SC‐DPC algorithm on phasor fluorescence data. It was selected for its widespread use for fluorescence‐data segmentation [22, 36]. It offers straightforward implementation, good interpretability of results, and robustness. In this study, k‐means was performed on phasor points representing fluorescence spectra from individual pixels.

### 3.5 Gaussian Mixture Model (GMM)

The Gaussian mixture model (GMM) [27] was used as a reference to evaluate the unmixing performance of the SC‐DPC algorithm. The GMM presents the distribution of data points in phasor space as a weighted sum of *n* multivariate Gaussian distributions. Unlike k‐means, which can assign only one spectral component to each pixel, the GMM is a probabilistic algorithm that describes intensity in each pixel as a weighted sum of all given spectral components. This approach enables a more accurate description of fluorophore composition in pixels where fluorophores exhibit strong spatial overlap. The GMM was also used previously for unmixing spectral phasor data [37]. In this study, the GMM was performed on phasor points representing fluorescence spectra from individual pixels. To ensure an unbiased comparison, the GMM was configured with the same number of components as SC‐DPC and implemented using the scikit‐learn python library [38]; initialization sensitivity was controlled by using the k‐means algorithm to establish initial cluster centers.

### 3.6 Statistical Analysis

To evaluate statistical robustness, all reported quantitative metrics (clustering/unmixing errors, including MAE and spectral parameter estimates) were computed across 10 independent dataset realizations per experimental condition. Unless specified otherwise, all values are presented as mean ± SD.

## 4 Results

### 4.1 Comparison of k‐Means and SC‐DPC Clustering on Synthetic Data

Our method was tested on synthetic data and compared with the k‐means algorithm. We have generated three datasets containing two fluorophores spread across 24 spectral channels, with different spectral separation between fluorophore peak wavelengths (Δ*λ* = 40, 30, and 20 nm). In each dataset, half the pixels contained intensity from both fluorophores, while the other half contained signal from only one. The spatial distribution of pixels with mixed contributions was random. Within each such pixel, fluorophore contributions were also assigned randomly, with the dominant fluorophore always contributing at least 50% of the total intensity. As outlined in Section 5 in SI, all reported values were obtained from 10 realizations of each dataset and reported as mean ± SD.

**FIGURE 3:**
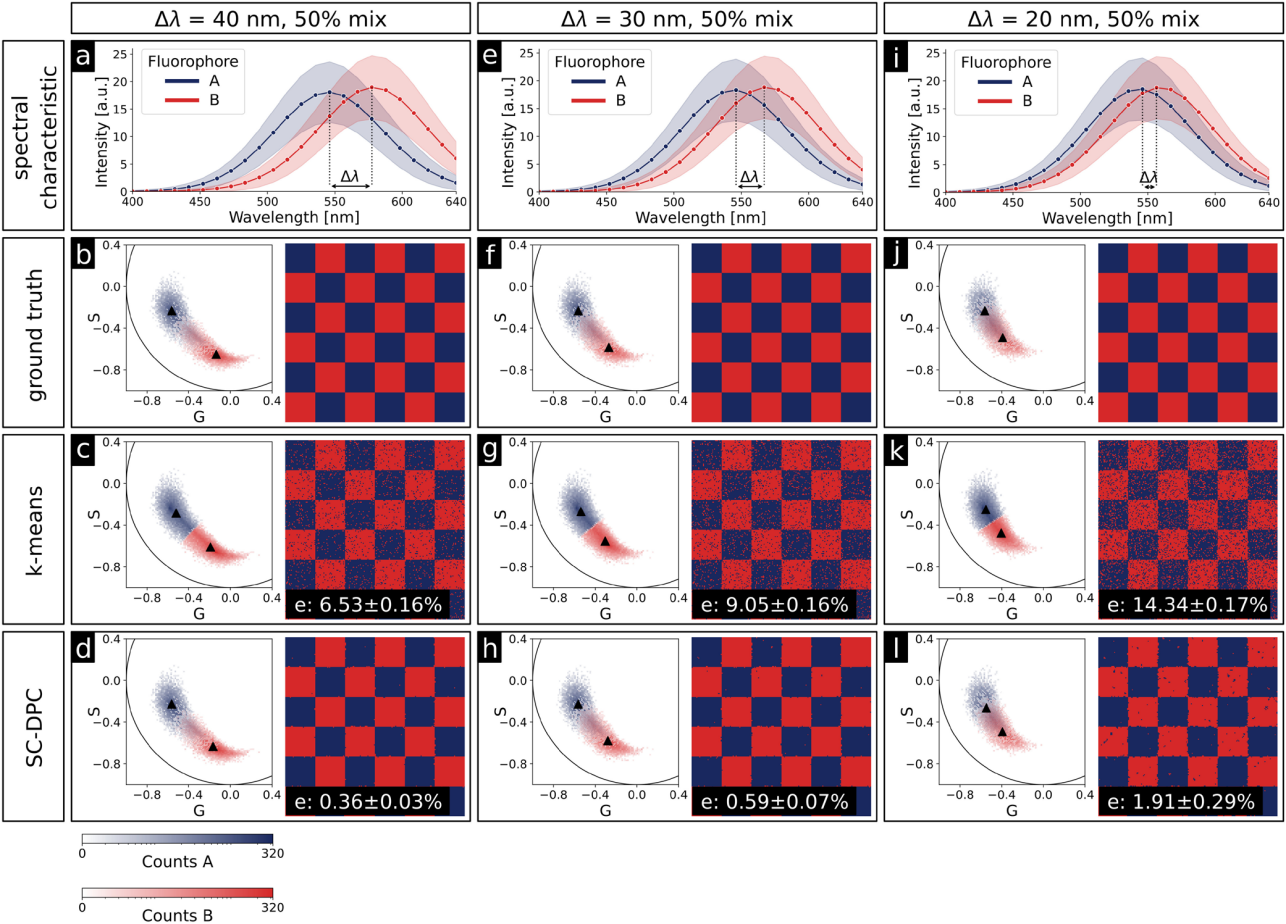
Comparison of k‐means and SC‐DPC clustering of synthetic data with two fluorophores. The two fluorophores were arrayed as 50% mixed and 50% pure pixels, under varying spectral separations. (a–d) Results are shown for spectral separations of 40 nm; (e–h) 30 nm; and (i–l) 20 nm. The first row displays the optical spectra of the fluorophores present in the dataset; the second row shows the ground‐truth phasor plot alongside the ground‐truth fluorophore spatial‐distribution pattern; in the third row, k‐means results for the phasor plot are shown, together with the reconstructed spatial pattern; and in the fourth row, the SC‐DPC results for the phasor plot are shown, along with the reconstructed spatial pattern. Each phasor plot contains two black‐triangle markers representing cluster centers. Each spatial‐pattern image contains a black box with the percentage of misidentified pixels (error) presented as mean ± SD.

Clustering results are presented in Figure 3. Panels (a–d) correspond to a spectral peak separation of 40 nm, (e–h) to 30 nm, and (i–l) to 20 nm. The first row shows the optical spectra of the fluorophores present in the image. The second row displays the ground‐truth phasor plots alongside the ground‐truth spatial patterns; the latter remained identical across datasets, despite differences in phasor distributions. Black triangles on the phasor plots indicate the cluster centers, defined as the mean positions of all points belonging to each cluster. Rows 3 and 4 of Figure 3 illustrate clustering results in phasor space and in the reconstructed spatial patterns, along with the pixel‐level estimation of error (percentage of pixels in which the dominant fluorophore was misidentified was determined across 10 independent realizations of each dataset, and is presented as mean ± SD). The effect of decreasing spectral separation, due to a shift in the peak wavelength of fluorophore B, is evident both in optical spectra and in the ground‐truth phasors (fluorophore A remains fixed). In the phasor plots, this dependence on spectral separation appeared as points dominated by fluorophore B moving toward the cluster of fluorophore A.

Across all tested conditions, SC‐DPC consistently outperformed k‐means in terms of clustering accuracy. The clustering accuracy was the highest for both algorithms when spectral separation was set to 40 nm (Figure 3a–d); however, SC‐DPC achieved excellent reconstruction accuracy (0.36% mean error) compared to 6.53% for k‐means. At 30 nm of separation (Figure 3e–h), the misclassification rate for k‐means rose to 9.05%, indicating difficulty in handling fluorophores with overlapping spectra. In contrast, SC‐DPC maintained robust accuracy with only 0.59% mean error, confirming that our method better resolved overlapping spectra. At the lowest spectral separation tested (20 nm, Figure 3i–l), the trend persisted: k‐means exhibited 14.34% error, while SC‐DPC exhibited only 1.91% mean error. Notably, SC‐DPC at 20 nm separation still outperformed k‐means at 40 nm, highlighting its superior ability to resolve overlapping spectral signatures. This nearly order‐of‐magnitude difference demonstrates that SC‐DPC provided superior separation of fluorophores under challenging conditions where emission spectra of fluorophores exhibit significant overlap. Overall, these results emphasize that while k‐means can identify fluorophores with well‐separated emission spectra, its clustering performance degrades significantly as spectral separation decreases. K‐means separates clusters with a linear boundary [39], which leads to substantial error in regions where clusters overlap. This overlap was caused by ~50/50 mixing in some pixels, and noise shifting points toward the opposite cluster. SC‐DPC was able to improve accuracy by incorporating spatial context and correcting pixel labels based on their neighborhood in the integrated intensity image. If a pixel was shifted towards the other cluster on the phasor plot but its neighbors were not, SC‐DPC was able to make the correction. For pixels with equal contributions from both fluorophores, a correct assignment was possible if neighboring pixels shared a dominant fluorophore. This spatial bias was weaker along the edges, resulting in higher error visible in Figure 3d,h,l. Incorporating spatial‐based correction into the method increased clustering accuracy, as shown by the black error boxes in Figure 3. These results were obtained for a dataset with the same SD of angular and radial noise for both fluorophores, which resulted in both fluorophores being represented by two clouds of points with similar spreads in phasor space. To test the behavior of k‐means and SC‐DPC in the presence of two fluorophores with different spreads, we generated an additional dataset with modified noise SDs. The results are presented in Figure S2.

K‐means and SC‐DPC estimate not only the intensity contributions, but also the cluster centers that correspond to the pure fluorophores. Each fluorophore is characterized by the spectral center of mass *λ*_*c*_ (angular position on the phasor plot) and spectral width *w*_*λ*_ (inversely related to radial position on the phasor plot). Table 1 presents values of cluster centers indicated by black triangles in Figure 3. Fluorophore A had centers of mass at 539.7, 538.6, and 537.5 nm and spectral widths of 98.0, 96.2, and 94.8 nm, while fluorophore B had centers of mass at 568.1, 560.2, and 552.0 nm with spectral widths of 87.5, 89.8, and 91.5 nm, respectively, for the different spectral separations. Presented values were calculated from generated phasor points of truncated spectra. Comparative evaluation shows that the k‐means achieved good accuracy in centers estimation across tested spectral separations. The estimates provided by SC‐DPC for both *λ*_*c*_ and *w*_*λ*_ were within uncertainty bounds, which are slightly bigger than for k‐means due to the binning during histogram analysis. Since detected centers are assigned in the middle of the bin, the uncertainty is equal to ± half of the bin's width in each axis (in this case, 0.4 nm during the fine search). These results indicated that SC‐DPC provides accurate centers estimation.

**TABLE 1:** Numerical comparison of true and estimated cluster centers generated for three datasets.

		Δ*λ* = 40 nm		Δ*λ* = 30 nm		Δ*λ* = 20 nm	
		*λ*_*c*_ (nm)	*w*_*λ*_ (nm)	*λ*_*c*_ (nm)	*w*_*λ*_ (nm)	*λ*_*c*_ (nm)	*w*_*λ*_ (nm)
Ground truth	Fluorophore A	539.7 ± 0.1	98.0 ± 0.1	538.6 ± 0.1	96.2 ± 0.1	537.5 ± 0.1	94.8 ± 0.1
	Fluorophore B	568.1 ± 0.1	87.5 ± 0.1	560.2 ± 0.1	89.8 ± 0.1	552.0 ± 0.1	91.5 ± 0.1
k‐means	Fluorophore A	539.1 ± 0.1	97.5 ± 0.1	537.8 ± 0.1	95.7 ± 0.1	536.2 ± 0.1	94.4 ± 0.1
	Fluorophore B	568.5 ± 0.1	86.5 ± 0.1	560.8 ± 0.1	88.7 ± 0.1	553.1 ± 0.1	90.0 ± 0.1
SC‐DPC	Fluorophore A	534.7 ± 0.2	94.3 ± 0.4	535.8 ± 0.2	94.3 ± 0.4	537.6 ± 0.2	94.3 ± 0.4
	Fluorophore B	571.2 ± 0.2	80.6 ± 0.4	562.9 ± 0.2	84.6 ± 0.4	554.2 ± 0.2	88.6 ± 0.4

*Note:* The individual datasets correspond to a fixed proportion of pixels with mixed contributions (50% containing contributions from both fluorophores, and 50% containing intensity from only one fluorophore), and varying spectral separations between fluorophore peak wavelengths (Δ*λ* = 40, 30, and 20 nm). Each value is reported as a mean ± SD calculated from 10 realizations of each dataset.

**FIGURE 4:**
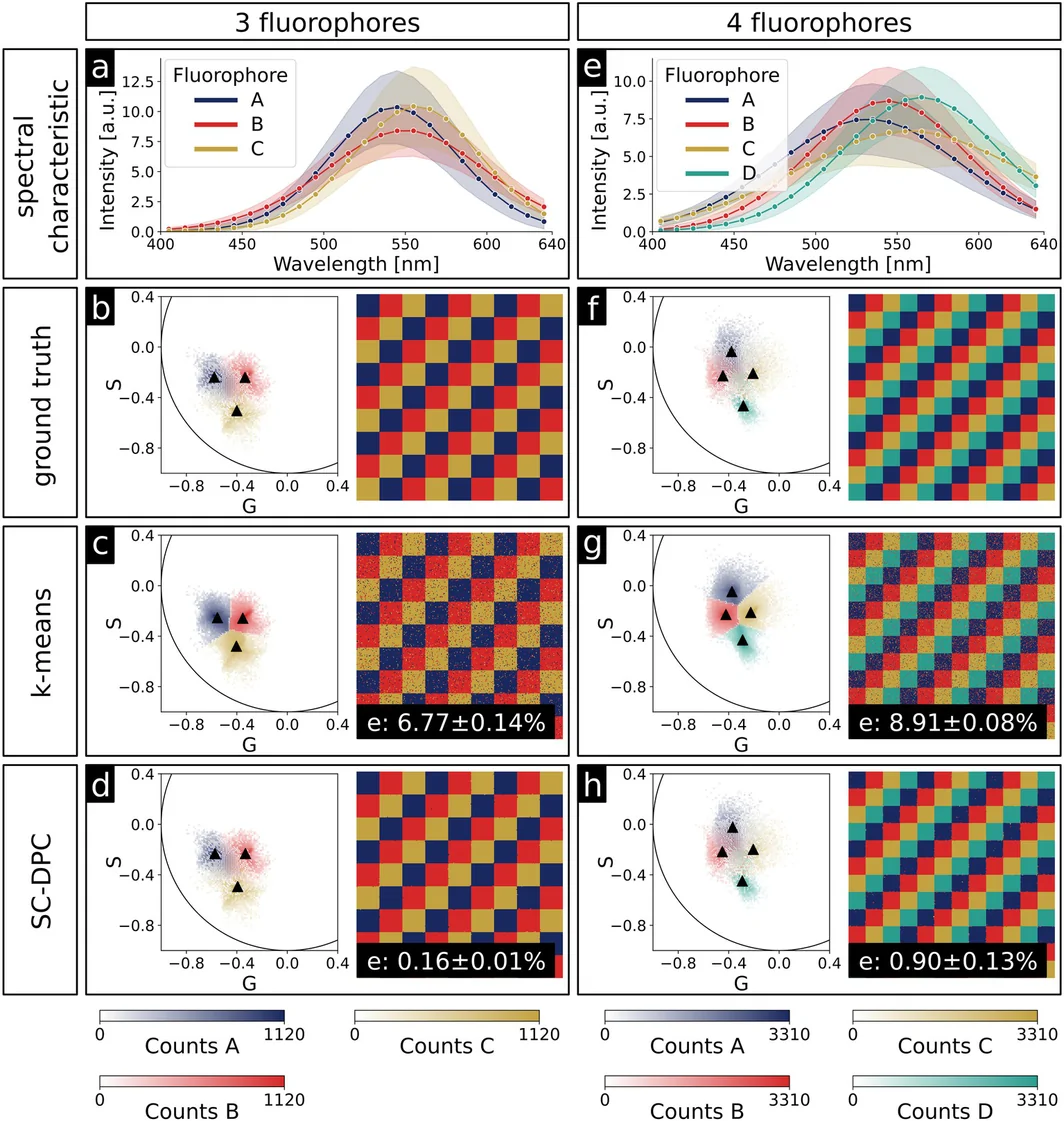
Comparison of k‐means and SC‐DPC clustering of synthetic data with three and four fluorophores. In each test, 50% of pixels from each dominant fluorophore contained mixed spectra. (a–d) Results are shown for a mixture of three fluorophores. Fluorophore A had a center of mass at 535.0 nm and a spectral width of 89.8 nm, fluorophore B had a center of mass at 543.8 nm and a spectral width of 140.9 nm, and fluorophore C had a center of mass at 554.4 nm and a spectral width of 85.5 nm. (e–h) Results are shown for a mixture of four fluorophores. Fluorophore A had a center of mass at 523.6 nm and a spectral width of 148.8 nm, fluorophore B had a center of mass at 538.1 nm and a spectral width of 119.8 nm, fluorophore C had a center of mass at 550.2 nm and a spectral width of 169.4 nm, and fluorophore D had a center of mass at 559.1 nm and a spectral width of 109.1 nm.

The performance of the SC‐DPC algorithm was further evaluated using a synthetic dataset containing three (Figure 4a–d) and four (Figure 4e–h) fluorophores overlapping both spectrally and spatially. The simulated spectra exhibit significant spectral overlap across the detection range (Figure 4a,e). Panels (b and f) show the ground‐truth phasor plot alongside the ground‐truth spatial pattern of dominant fluorophores. Panels (c, g) and (d, h) illustrate clustering results in phasor space and the reconstructed spatial pattern, along with the pixel‐level estimation error (mean ± SD), for k‐means and SC‐DPC, respectively. As presented, SC‐DPC still outperformed k‐means in a harsher environment, with an increased number of fluorophores and strong spectral and spatial overlap.

When dealing with multiple fluorophores, a low‐abundance component can easily be obscured or lost. To evaluate the performance of our method (particularly the Gaussian smoothing step of the phasor plot) in such scenarios, we generated an additional dataset featuring three fluorophores, where one was significantly less abundant than the rest. The less abundant fluorophore was dominant in only around 1.2% of all pixels and exhibited substantial spectral and spatial overlap with other components, enabling a test of the performance of SC‐DPC in the presence of a minor spectral population. The results presented in Figure S3 show that Gaussian smoothing did not eliminate the minor peak in the phasor plot. Despite its low abundance, the peak was detected after filtering, confirming that the smoothing procedure preserves sparse but distinct spectral features. In this test, SC‐DPC successfully identified all three fluorophores and misassigned only 1.31% of all pixels on average, whereas k‐means did not recognize the low‐abundance cluster properly, instead placing it between the clusters corresponding to the high‐abundance spectral components, increasing clustering error to 60.19% on average. These results confirm that the proposed approach remains robust in the presence of a minor population.

### 4.2 Comparison of GMM and SC‐DPC Unmixing of Synthetic Data

While the previous section focused on the clustering capabilities of SC‐DPC, the algorithm also provides quantitative information about the intensity contributions of each fluorophore. To evaluate this unmixing performance, we compared SC‐DPC with the GMM, since k‐means does not provide the quantitative information. The unmixing performance of both algorithms was evaluated using the mean absolute error (MAE) calculated for the reconstructed intensity contributions versus the known ground‐truth values. The definition of the metric is provided in the Supporting Information. Unmixing was performed on a previously used dataset containing three fluorophores (Figure 4a,b), with the number of mixed pixels increased from 50% to 100%.

**FIGURE 5:**
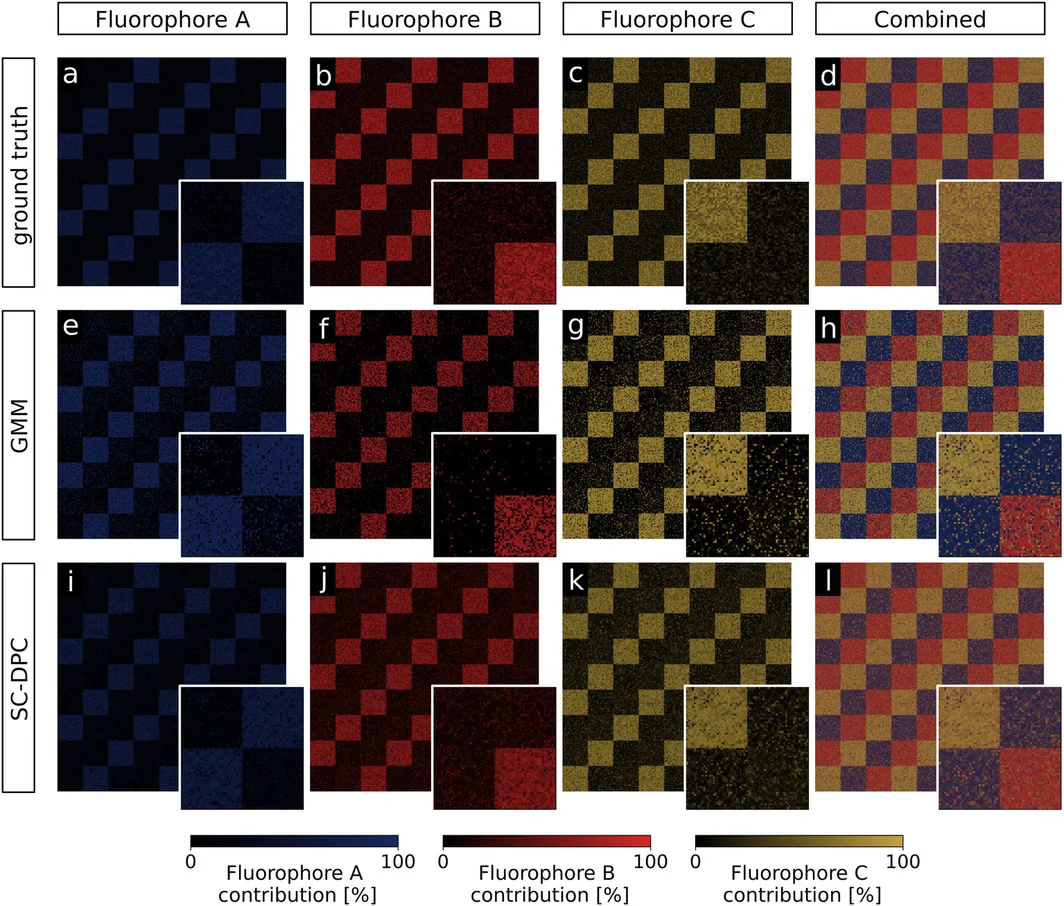
Comparison of unmixing results obtained with the GMM and SC‐DPC. (a–d) Ground truth of intensity contributions from individual fluorophores and combined intensity image. (e–h) Intensity contributions from individual fluorophores, along with the combined intensity image obtained with GMM. (i–l) Intensity contributions from individual fluorophores, along with the combined intensity image obtained with SC‐DPC. Each intensity image is paired with a zoomed‐in section of its bottom‐right corner.

The unmixing results are presented in Figure 5. Each row displays the individual intensity contributions for the three fluorophores, followed by an image representing a combination of the individual contributions. The first row presents ground‐truth contributions (Figure 5a–d), the second row presents reconstructed intensity images obtained with the GMM (Figure 5e–h), and the third row shows results for SC‐DPC (Figure 5i–l). Each image is paired with a zoomed‐in section of its bottom‐right corner, allowing comparison of fine details between the images. The most significant difference between the GMM and SC‐DPC reconstructions can be observed in the zoomed‐in sections. The GMM produces rather sparse images, where most pixels have either a high‐ or low‐intensity contribution, with many high‐value contribution pixels leaked into the areas dominated by other fluorophores. In contrast, SC‐DPC creates smooth images with high‐contribution values assigned to the areas with dominant fluorophores and low values to other areas. Compared to ground truth, SC‐DPC performs better than the GMM as it confines high contributions within the regions related to the dominant fluorophores.

The visual presentation of results is supported by the quantitative error metrics. The MAE, which represents the average percentage of misassigned intensity between fluorophores per each fluorophore in each pixel, was found to be 15.33% ± 0.22% for the GMM, and only 10.20% ± 0.25% for SC‐DPC. The lower value of MAE shows that SC‐DPC effectively reduced estimation error and suggests that the spatial correction implemented in SC‐DPC is efficient at suppressing significant misestimations. Collectively, these results demonstrate that by incorporating spatial context, SC‐DPC provides more accurate unmixing, outperforming the probabilistic GMM approach in reconstructing partial intensities related to individual fluorophores.

### 4.3 SC‐DPC Clustering and Unmixing of Experimental Data: Two‐Photon Excited‐Fluorescence Imaging of Mouse Retina

**FIGURE 6:**
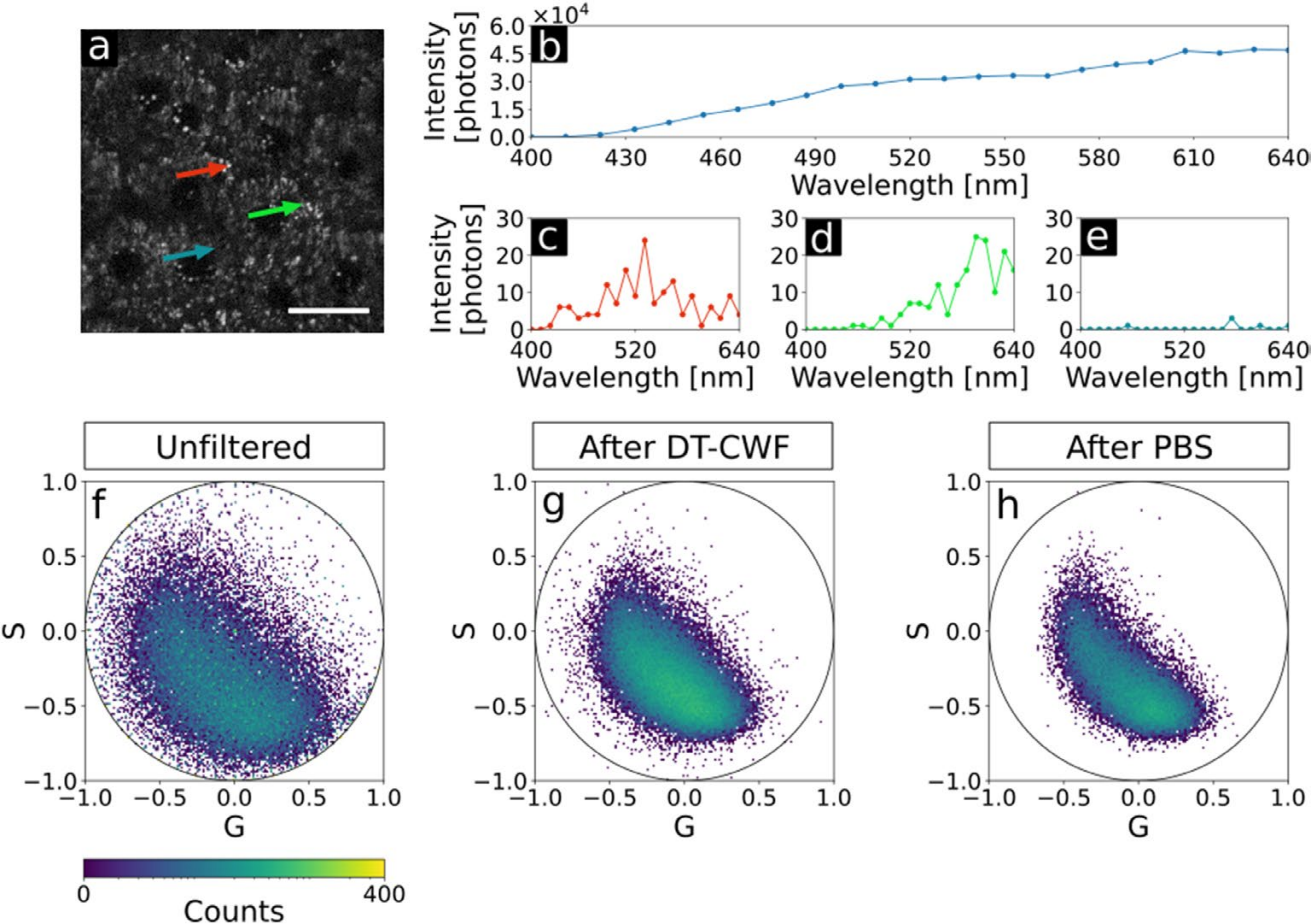
Visualization of experimental data. (a) Integrated intensity image with gamma correction (gamma = 2); scale bar, 20 μm. (b) Integrated spectrum of the entire image. (c–e) Example spectra obtained from individual pixels. Spectral phasor plots of experimental data are shown after several analytical operations: After phasor transformation (f); after DT‐CWF (g); and after PBS (h). The logarithmic color scale indicates the number of phasor points located in each color‐coded area.

After testing the two methods on synthetic datasets, we applied them to a two‐photon hyperspectral image of mouse RPE cells. Figure 6a shows the integrated intensity image with gamma correction applied to enhance visibility. *Abca4* ^−/−^ *Rdh8* ^−/−^ mice exhibit pronounced accumulation of retinoid‐derived bisretinoids, including A2E, as well as excess retinyl esters, resulting in markedly elevated RPE autofluorescence relative to wild‐type animals [33]. Individual RPE cells form a mosaic of darker and lighter regions, while numerous small, highly fluorescent cytoplasmic granules are dispersed throughout the layer. These discrete puncta correspond to retinosomes, which are retinyl‐ester–rich storage bodies that generate strong intrinsic fluorescence under two‐photon excitation in this mouse model [23].

The integrated spectrum of the entire image presented in Figure 6b exhibits two peaks centered near 540 and 640 nm. These peaks are consistent with the expected emission of retinyl esters and A2E, respectively; however, more precise analysis is challenging due to substantial spectral overlap. Example spectra from individual pixels are presented in Figure 6c–e. The first one was registered in one of the bright granules with a center of mass around 530 nm, the second one was registered in another bright region with a center of mass near 610 nm, and the third one was registered in a low‐intensity region.

We started analyzing data by performing phasor transformation within 400–640 nm on fluorescence spectra from individual pixels, which resulted in the phasor plot presented in Figure 6f, where points create a diffuse cloud with many high‐density artifacts. After filtering with DT‐CWF, the phasor plot in Figure 6g displays a visibly improved point distribution, with greater conciseness and without artifacts. Removing phasor points representing pixels labeled as background by PBS further improved the conciseness of the phasor points, which can be observed in Figure 6h. In our dataset, 49.4% of pixels were identified as containing only background. The histogram of the intensity distribution of the pixel is shown in Figure S4, together with spectral analysis of the pixels assigned as signal and background.

**FIGURE 7:**
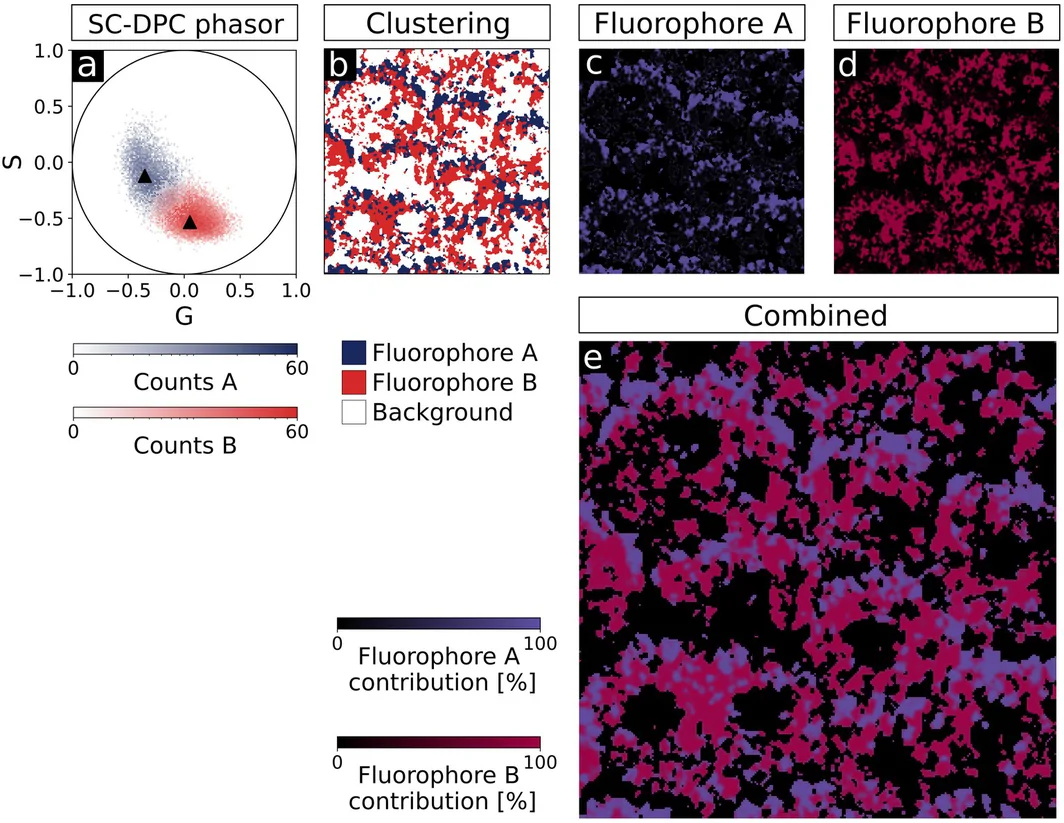
SC‐DPC clustering and unmixing results for experimental data. (a) Phasor plot colored according to the dominant fluorophores obtained via SC‐DPC clustering. (b) Spatial distribution of the dominant fluorophores obtained via SC‐DPC clustering. (c, d) Images presenting intensity contributions of fluorophores A and B, obtained via SC‐DPC unmixing scaled as percentage of total intensity. (e) Combined image containing intensity contributions from both unmixed fluorophores presented in panels (c and d). The logarithmic color map on the phasor plot indicates phasor‐point density, with blue representing fluorophore A and red representing fluorophore B.

Figure 7 presents clustering and unmixing results obtained with SC‐DPC for the experimental data [23]. Figure 7a shows a spectral phasor plot with color‐coded clusters and phasor‐point counts indicated by color intensity. The spatial distribution of the dominant fluorophores obtained via clustering is presented in Figure 7b. Both fluorophores are marked with their respective colors, while the background is marked with white. Unmixing results are presented in Figure 7c,d, which show estimated intensity contributions of both fluorophores scaled as a percentage of each pixel's total intensity. Figure 7e presents a combined image of both fluorophore‐intensity estimations.

SC‐DPC produced two partially overlapping clusters in the phasor plot, reflecting the smooth transitions between fluorophores. The cluster centers estimated by SC‐DPC placed fluorophore A at a spectral center of mass of 532.7 nm with a width of 150.7 nm, while fluorophore B was estimated with a center of mass at 583.6 nm and a width of 111.2 nm. Clustering results were used to plot a spatial distribution of both fluorophores, showing where each fluorophore contributed at least 50% of intensity. As presented in Figure 7b, fluorophore A was mostly present around the cell borders, while fluorophore B tended to accumulate inside. Unmixing results can be used for further analysis of the sample, as they provide information not only about the dominant fluorophores, but also about their contributions across the entire image. These estimated contributions, scaled as the percentage of each pixel's total intensity, were plotted in Figure 7c,d, with their combination presented in Figure 7e. The calculated contributions can also be used to obtain intensity images scaled as number of photons, which are presented in Figure S5.

## 5 Discussion and Summary

In this study, we introduced the SC‐DPC algorithm, a novel method that addresses the challenges of clustering fluorescence spectra in phasor space in the presence of fluorophores that exhibit significant spectral overlap. Together with SC‐DPC, we proposed a workflow that removes noise and background, improving data quality and reducing data volume by excluding background pixels from the clustering. SC‐DPC was compared with the k‐means algorithm in terms of clustering accuracy. Across tests on synthetic data containing two fluorophores, SC‐DPC achieved clustering errors 8‐ to 16‐times lower than those of k‐means, while maintaining comparable precision in center estimation. Additional evaluation on datasets containing more fluorophores showed that SC‐DPC can reduce the clustering error up to 39 times compared to k‐means. We further tested both methods on one more dataset with uneven cluster spreads. The test revealed that k‐means introduced a clear bias toward one fluorophore, while SC‐DPC performed similarly to previous tests, with most errors occurring around the edges. We also evaluated SC‐DPC unmixing performance against the GMM, which showed that on average SC‐DPC reduced high‐value misestimations but slightly increased the low‐value ones. We also demonstrated the utility of SC‐DPC in segmentation of experimental data, which produced results aligned with previous findings, although a quantitative evaluation against other algorithms was not possible due to the lack of ground truth. These results demonstrate that SC‐DPC shows markedly improved performance for clustering of phasor data under challenging conditions. The robustness of SC‐DPC in scenarios involving substantial spectral overlap between fluorophores and uneven cluster distributions can improve the reliability of data segmentation in fluorescence microscopy. Moreover, the algorithm is compatible with existing phasor‐analysis frameworks, potentially enabling its integration into a broad range of applications based on hyperspectral imaging or fluorescence‐lifetime imaging microscopy (FLIM), including the analysis of metabolic states and Förster resonance energy transfer (FRET) [40, 41, 42].

A key feature of the SC‐DPC phasor analysis is its reliance on the first harmonic (*n* = 1) for clustering and fluorophore separation. While higher‐order harmonics can theoretically capture finer spectral details, the first harmonic provides the highest signal‐to‐noise ratio (SNR) and remains the gold standard for robust spectral discrimination in most experimental settings [17, 18, 20]. Furthermore, prioritizing the first harmonic ensures that our computational framework remains directly compatible with emerging hardware‐based phasor technologies. It has been demonstrated that the phasor transform can be performed physically (in the optical path) using a pair of sine and cosine filters [43]. This approach bypasses the need for full hyperspectral cube acquisition, significantly reducing data overhead and enabling faster processing [17]. Such hardware‐implemented phasor systems have proven highly effective for high‐speed imaging [18] and have been successfully translated to common microscopy platforms for practical biological applications [44]. By optimizing our clustering algorithm for *n* = 1, we demonstrate its utility not only for 24‐channel hyperspectral data but also for simplified high‐speed systems that utilize specialized optical filters. Focusing on the first harmonic ensures that the method is scalable and broadly applicable across different imaging modalities. Nonetheless, the mathematical architecture of our clustering stage is *n*‐dimensional; it can be extended readily to include higher harmonics (*G*_*n*_, *S*_*n*_) for specific applications where extreme spectral overlap necessitates additional discriminative dimensions at the cost of increased noise sensitivity.

The proposed spatial‐correction term operates on the principle of local spectral similarity. In practical fluorescence microscopy, imaging parameters are typically selected so that pixels are significantly smaller than the biological structures of interest, ensuring a degree of spectral similarity between neighboring pixels and making isolated single‐pixel features exceptionally rare. Even for small structures like the retinosomes shown in the experimental dataset, features span multiple pixels, allowing SC‐DPC to segment them reliably based on distinct spectral properties without losing fine spatial features. Conducted tests demonstrated that SC‐DPC maintains a significant accuracy advantage over methods like k‐means or GMM that do not use local similarity of spectra, even when local spectral similarity extends across only a couple of pixels.

SC‐DPC has the potential to be computationally efficient, with shorter execution times than k‐means in all the tested scenarios. This performance advantage stems from the non‐iterative architecture of the algorithm. While processing time of k‐means scales with the number of pixels, for SC‐DPC it scales primarily with the number of histogram bins. Although precise numbers are difficult to report at this stage, rough estimates indicate that SC‐DPC can perform clustering faster than k‐means in every tested scenario, making it potentially useful for real‐time applications and processing of large images, where algorithms such as k‐means exhibit lower efficiency [45].

Despite its advantages, SC‐DPC has some limitations. The number of fluorophores in the sample must be known a priori. Additionally, the histogram bin counts should be adjusted to spectral channel width and the smallest separation between the peaks (if known). The remaining parameters, including the Gaussian smoothing kernel, the k parameter in PBS, and the number of decomposition levels in DT‐CWF, can be modified to change the density peak detection behavior or adjust filtering strength, but they remain fixed under standard operation. Furthermore, the method should be validated on a larger set of experimental datasets with semi‐ground truth available for quantitative evaluation. We also plan to explore applications of SC‐DPC in FLIM imaging, as well as improvements to the automation of the entire workflow.

In conclusion, SC‐DPC offers a robust and computationally efficient solution for spectral phasor data analysis. It matches the well‐established k‐means algorithm in center estimation and consistently outperforms k‐means in estimating dominant fluorophores. SC‐DPC can also be used to estimate intensity contributions, achieving higher accuracy than the GMM. These characteristics make SC‐DPC a promising algorithm for fluorescence imaging, large‐scale data analysis, and future real‐time applications.

## Funding

This study was funded in full by the National Science Centre (NCN, Poland), grant number 2021/43/D/ST7/01126 and National Institutes of Health, P30EY034070.

## Conflicts of Interest

M.B. and J.B. declare that a disclosure of invention related to the research presented in this manuscript has been submitted to the Wrocław Centre for Technology Transfer at Wrocław University of Science and Technology. The other authors declare no conflicts of interest.

## Supporting information


**Figure S1:** Example of a synthetic dataset without spectral truncation. (a) Statistical distribution of the generated spectra for the two fluorophores, where solid lines denote the mean intensity and shaded regions represent one SD (spectral separation is set to 27 nm; 50% of pixels contain mixed intensity contributions); (b) phasor representation of the dataset, where each point is marked with a color corresponding to the dominant fluorophore. Black triangles indicate the cluster centers, defined as the mean positions of all points belonging to the cluster.
**Figure S2:** Influence of uneven phasor‐points distribution on spatial‐distribution error. Comparisons are presented on the phasor plots and error spatial distribution masks, with errors marked in white. (a) Reference clustering results on the phasor for k‐means. (b) Reference error mask for k‐means; uniform error distribution. (c) Reference clustering results on phasor for SC‐DPC. (d) Reference error mask for SC‐DPC results. (e) Clustering results on the phasor for k‐means; substantial error occurs due to uneven distribution. (f) Error mask for k‐means results; biased error appears toward one of the fluorophores. (g) Clustering results on the phasor for SC‐DPC. (h) Error mask for the SC‐DPC results; errors are located mostly around the edges due to a lack of strong correcting bias.
**Figure S3:** Comparison of k‐means and SC‐DPC clustering on synthetic data with a minor spectral population. Fluorophore A had a center of mass at 517.0 nm and a spectral width of 123.0 nm, fluorophore B had a center of mass at 540.8 nm and a spectral width of 143.0 nm, and fluorophore C had a center of mass at 561.3 nm and a spectral width of 127.2 nm. In this dataset, 50% of pixels from each dominant fluorophore contained mixed spectra. (a) Spectral characteristics of generated fluorophores. (b) Ground truth phasor plot, alongside the ground truth fluorophore spatial distribution pattern. (c) K‐means results on the phasor plot, alongside reconstructed spatial distribution. (d) SC‐DPC results on the phasor plot, along with the reconstructed spatial pattern. Each phasor plot contains three black‐triangle markers representing cluster centers. Each reconstructed spatial pattern contains a black box with the percentage of misidentified pixels (error) presented as mean ± SD.
**Figure S4:** Analysis of PBS influence on spectral distribution. (a) Histogram of the total pixel intensities within the dataset. Blue color marks low‐intensity (less than six photons) pixels labeled as background. Orange color marks pixels labeled as signal (six photons per pixel or more). (b) Average photon counts per pixel across spectral channels for three groups: background, signal, and all pixels in the dataset.
**Figure S5:** SC‐DPC unmixing results for experimental data scaled in photon counts. (a, b) Images presenting intensity of two fluorophores obtained via SC‐DPC unmixing scaled in photon counts. (c) Combined image containing intensity from both unmixed fluorophores presented in panels a and b together with the low‐level background. Presented intensity images were enhanced using gamma correction due to low photon counts.

## Data Availability

The data that support the findings of this study are openly available in RepOD Repozytorium Otwartych Danych at https://doi.org/10.18150/9VTEZ6 [46].
