# Supplementary material for: Phasor‐Based Spatio‐Spectral Segmentation for Hyperspectral Fluorescence Microscopy
Source: J Biophotonics. 2026 Sep 3;19(9):e70351. doi: 10.1002/jbio.70351 (PMC13541396; doi:10.1002/jbio.70351)
Supplement: Supplementary file 1 [file JBIO-19-e70351-s001.docx]

Phasor-based spatio-spectral segmentation for hyperspectral fluorescence microscopy

**1 Description of density peak searching algorithm**

The SC-DPC algorithm consists of three steps, the first one being a density peak detection in the histogram created from a phasor plot. For this task, we used a modified version of a graph-based algorithm described in [32]. In this method, each histogram bin is treated as a node, and neighboring bins are connected through parent–child relationships based on the number of phasor points they contain. Each bin becomes a child of its neighbor with a higher number of points, and bins without a parent are identified as density peaks. In case of noisy data, the algorithm may identify too many peaks. To stabilize this process, we implemented a coarse-to-fine search strategy.

Initially, a coarse search is performed on a histogram with a relatively small number of bins to locate approximate regions where density peaks may be present. If the coarse search identifies more peaks than expected, Gaussian smoothing is applied to the histogram as a low-pass filter to remove local spurious peaks caused by noise or artifacts. Importantly, peaks are not removed solely due to the low quantity of phasor points. There is a trade-off between peak strength and proximity to neighboring density peaks. Minor peaks that are well separated from dominant peaks are preserved, ensuring that low-abundance but distinct spectral components remain represented. The smoothing kernel was adopted from [32] without modification, as it was designed for the removal of spurious peaks while retaining meaningful spectral components. In the presence of a significant, unmodeled cluster whose peaks cannot be smoothed, an additional mismatch check safely terminates the run and prompts the user to increment n, preventing the omission of major clusters. Crucially, because all peaks are identified first and discarded later, an incorrect n does not corrupt the centers related to expected *n* clusters.

Once the expected number of coarse regions is identified, the algorithm proceeds to the fine search. Each coarse region is divided into smaller bins, and a second, high-resolution search is performed to find the precise peak locations. Using a coarse-to-fine approach can potentially increase search efficiency by investigating smaller number of bigger bins in the coarse search and then increasing search resolution in a small number of bins, that have been determined to contain a density peak. Furthermore, this approach ensures robust operation even in highly mixed datasets lacking sharp density peaks; the low-resolution step initially encompasses the diffuse clusters in a small number of bins to find their broad centers of mass, preventing noise-induced misestimation during the subsequent high-resolution refinement. This procedure is performed on the histogram rather than individual phasor points, ensuring preservation of the linear mixing model inherent to phasor analysis.

**2 Synthetic dataset without spectral truncation**

Synthetic datasets used for the evaluation of SC-DPC exhibit significant spectral truncation, which leads to the formation of elongated tails towards longer wavelengths in the phasor plot. We have generated an additional dataset without spectral truncation to confirm our model (**Fig. S1a**). The resulting phasor distribution follows the expected behavior (**Fig. S1b**), confirming that the elongated tails observed in the synthetic phasor data are caused by the presence of spectral truncation.

**Fig. S1. Example of a synthetic dataset without spectral truncation.** (**a**) Statistical distribution of the generated spectra for the two fluorophores, where solid lines denote the mean intensity and shaded regions represent one SD (spectral separation is set to 27 nm; 50% of pixels contain mixed intensity contributions); (**b**) phasor representation of the dataset, where each point is marked with a color corresponding to the dominant fluorophore. Black triangles indicate the cluster centers, defined as the mean positions of all points belonging to the cluster.

**3 Analysis of clustering errors in k-means and SC-DPC**

The synthetic datasets containing two fluorophores presented in Fig. 3, were generated with the same SD of angular and radial noise for both fluorophores, resulting in two clouds of points with similar spreads in phasor space. To test the behavior of k-means and SC-DPC in the presence of two fluorophores with different spreads, we generated an additional dataset with modified noise SDs. The comparison was performed on a dataset with 30 nm of spectral separation and 50% of pixels containing mixed spectra, previously used in tests presented in Fig. 3. Reference phasor plots are shown in the first row of **Fig. S2**, supplemented by spatial error distribution patterns, with misclassified pixels marked in white. Panels a and b show the clustered phasor plot and spatial-error distribution for k-means. Panels c and d show results for SC-DPC. The second row of Fig. S2 presents results obtained using data with modified noise distributions, with results for k-means shown in panels e and f, and for SC-DPC in panels g and h.

***Fig. S2. Influence of uneven phasor-points distribution on spatial-distribution error.*** *Comparisons are presented on the phasor plots and error spatial distribution masks, with errors marked in white. (****a****) Reference clustering results on the phasor for k-means. (****b****) Reference error mask for k-means; uniform error distribution. (****c****) Reference clustering results on phasor for SC-DPC. (****d****) Reference error mask for SC-DPC results. (****e****) Clustering results on the phasor for k-means; substantial error occurs due to uneven distribution. (****f****) Error mask for k-means results; biased error appears toward one of the fluorophores. (****g****) Clustering results on the phasor for SC-DPC. (****h****) Error mask for the SC-DPC results; errors are located mostly around the edges due to a lack of strong correcting bias.*

In the test with equal SDs of errors, k-means misidentified 9.05% of all pixels on average. The clustered phasor plot is shown in **Fig. S2a**, and the spatial distribution of misidentified pixels shown in **Fig. S2b** reveals their uniform distribution. SC-DPC misidentified 0.59% of pixels on average, creating two overlapping clusters in the phasor plot (**Fig. S2c**). The misidentified pixels were located near the edges of the structures, as shown in **Fig. S2d**.

Introducing unequal SDs of errors altered the spread of both clusters. **Fig. S2e** shows the clusters produced by k-means under these conditions. In this case, k-means misidentified 6.15% (± 0.08%) of all pixels; however, the error distribution was no longer uniform. In one example of dataset realizations, 2066 pixels with dominant fluorophore A were incorrectly assigned to fluorophore B, while only 179 pixels were misassigned in the opposite direction. This imbalance led to significant degradation of the structure associated with fluorophore A, as shown in **Fig. S2f**. SC-DPC misidentified 0.52% (± 0.05%) of pixels and created two overlapping clusters, accounting for the differing spreads within each cluster (**Fig. S2g**). The spatial distribution of misidentified pixels, shown in **Fig. S2h**, closely resembled that observed in Fig. S2d. Most misidentifications in the same example dataset occurred near the edges of the chessboard pattern, with 193 of the 223 misidentified pixels located along the edges, resulting in no additional degradation of the chessboard structure compared to the test case with equal SDs of errors.

The clustering errors produced by k-means and SC-DPC differed markedly in their spatial distributions, reflecting the underlying principles of each method. As described previously, k-means separates clusters using a linear boundary positioned in the middle between cluster centers in phasor space. As a result, k-means errors are typically concentrated around the boundary in phasor space, where the cluster centers cannot be clearly separated. The SC-DPC algorithm uses neighboring pixels in the integrated intensity image to correct calculated intensity contributions, thereby reducing clustering error in the area between the cluster centers in phasor space. However, this correction requires a strong bias toward one of the clusters. In our tests, SC-DPC misidentifications accumulated along the borders between regions dominated by different fluorophores in the integrated intensity image, where the bias needed for correction was weaker. The property of reducing clustering error in regions dominated by a single fluorophore exhibited by the SC-DPC algorithm resulted in less obstruction of the structures and smoother images, enabling a more accurate representation of the imaged sample.

**4 Performance evaluation on the dataset with minor spectral population**

To evaluate whether Gaussian smoothing of the phasor density histogram, created from a phasor plot (phasor histogram), leads to the loss of low-abundance spectral components, we generated a synthetic dataset containing three fluorophores with one significantly less abundant than the others. The less abundant fluorophore was dominant in only around 1.2% of all pixels and had an average intensity of 30 photons per pixel (compared to 100 photons of average intensity for the other two fluorophores). Generated fluorophores exhibited substantial spectral and spatial overlap with each other (**Fig. S3a**), allowing to test the ability of SC-DPC to preserve and accurately identify low-abundance components. **Figure S3b** shows the ground truth phasor plot alongside the ground truth spatial pattern of dominant fluorophores. Black triangles on the phasor plot indicate the cluster centers defined as the mean positions of all points belonging to each cluster. **Figures S3c** and **S3d** illustrate clustering results in phasor space and the reconstructed spatial pattern for k-means and SC-DPC, respectively. The percentage of pixels in which the dominant fluorophore was misidentified is also indicated in the figure. Gaussian smoothing of the phasor density histogram was applied during the peak detection as a part of the SC-DPC workflow. As presented, it did not eliminate the minor peak related to the low-abundance fluorophore. The smoothing kernel was chosen appropriately to balance the removal of spurious peaks and preservation of the significant but low-density peaks. As a result, the implemented smoothing acts as a low-pass filter that is well-suited for processing phasor density histograms. As presented, SC-DPC outperformed k-means in the presence of a minor spectral population, misassigning only 1.31% of all pixels on average. In contrast, k-means failed to recover the minor cluster and instead placed it between the clusters corresponding to the high-abundance spectral components, increasing clustering error to 60.19% on average. These results demonstrate that histogram-based peak detection with Gaussian smoothing enables reliable clusterization of meaningful spectral components even when they are weakly represented.

**Fig. S3. Comparison of k-means and SC-DPC clustering on synthetic data with a minor spectral population.** Fluorophore A had a center of mass at 517.0 nm and a spectral width of 123.0 nm, fluorophore B had a center of mass at 540.8 nm and a spectral width of 143.0 nm, and fluorophore C had a center of mass at 561.3 nm and a spectral width of 127.2 nm. In this dataset, 50% of pixels from each dominant fluorophore contained mixed spectra. (**a**) Spectral characteristics of generated fluorophores. (**b**) Ground truth phasor plot, alongside the ground truth fluorophore spatial distribution pattern. (**c**) K-means results on the phasor plot, alongside reconstructed spatial distribution. (**d**) SC-DPC results on the phasor plot, along with the reconstructed spatial pattern. Each phasor plot contains three black-triangle markers representing cluster centers. Each reconstructed spatial pattern contains a black box with the percentage of misidentified pixels (error) presented as mean ± SD.

**5 Definition of mean absolute error (MAE) used for quantitative evaluation of unmixing performance**

To quantitatively evaluate the accuracy of the intensity contributions estimation achieved by GMM and SC-DPC, we calculated the mean absolute error (MAE) for each fluorophore in a single pixel. The MAE represents the average misestimation between the ground truth intensity and the estimated intensity for each fluorophore at pixel *n*, normalized by the total pixel intensity:

$$\begin{aligned} MAE=\frac{1}{N\cdot K}\sum_{n=1}^{N} \sum_{k=1}^{K} \frac{\left| I_{n,k}-\hat{I_{n,k}} \right|}{I_{n}},\#\left( 1 \right) \end{aligned}$$

where *N* is the total number of pixels, *K* is the number of fluorophores, *I*_n,k_ and *î*_n,k_ are the ground truth and estimated intensity contributions of fluorophore *k* in pixel *n* respectively and *I*_n_ is the total intensity in pixel *n*. This metric effectively captures the average percentage of a single fluorophore's intensity that is misassigned to another fluorophore per pixel. A lower MAE indicates a more accurate reconstruction of the fluorophores contributions.

**6 Evaluation of the PBS influence on the spectral distribution in experimental data**

To assess the effect of Poisson Background Separation (PBS) on the spectral distribution in low-photon-count experimental data, we analyzed the intensity histogram of the experimental dataset together with a plot showing the mean number of photons per pixel by spectral channel. The histogram of total pixel intensities presented in **Fig. S4a** shows the separation between background and signal, with the threshold determined automatically based on Poisson statistics. In the presented experimental dataset, pixels containing less than six photons were labeled as background. To verify that this thresholding does not bias the spectral shape and phasor distribution, we compared the average recorded spectra for all pixels against those labeled as “signal” and “background” (**Fig. S4b**). The spectral profile of the signal pixels closely matches that of the total pixel population, indicating that the underlying spectral distribution remains unchanged. The primary effect of PBS is the removal of the lowest-intensity pixels.

**Fig. S4. Analysis of PBS** **influence on spectral distribution.** (**a**) Histogram of the total pixel intensities within the dataset. Blue color marks low-intensity (less than six photons) pixels labeled as background. Orange color marks pixels labeled as signal (six photons per pixel or more). (**b**) Average photon counts per pixel across spectral channels for three groups: background, signal, and all pixels in the dataset.

**7 Intensity images obtained via SC-DPC unmixing**

The unmixing results presented in Fig. 8c–e were scaled in percentages of total pixel intensity to focus on the spatial distribution of both fluorophores and their relationship. However, these results can also be expressed as a number of photons, to show the intensity of both fluorophores and how they mix. **Figures S5a** and **b** show fluorophore A and B contributions multiplied by the integrated intensity image, which focus on how many photons were emitted by each fluorophore, regardless of their proportions. Additionally, **Fig. S5c** presents their combination together with the low-level background that was previously filtered out using PBS.

**Fig. S5. SC-DPC unmixing results for experimental data scaled in photon counts.** (**a, b**) Images presenting intensity of two fluorophores obtained via SC-DPC unmixing scaled in photon counts. (**c**) Combined image containing intensity from both unmixed fluorophores presented in panels a and b together with the low-level background. Presented intensity images were enhanced using gamma correction due to low photon counts.
